# Reference values for fatigued versus non-fatigued limb symmetry index measured by a newly designed single-leg hop test battery in healthy subjects: a pilot study

**DOI:** 10.1007/s11332-017-0410-5

**Published:** 2017-11-10

**Authors:** Iris Leister, Georg Mattiassich, Harald Kindermann, Reinhold Ortmaier, Jürgen Barthofer, Imre Vasvary, Klaus Katzensteiner, Christine Stelzhammer, Stefan Tino Kulnik

**Affiliations:** 1 Department of Health Sciences, FH Campus Wien-University of Applied Sciences, Vienna, Austria; 2 Physiotherapie Linz Artmann & Scheibelberger, Salzburgerstraße 205, 4020 Linz, Austria; 3 Trauma Center Linz, Teaching Hospital of the Paracelsus Medical University Salzburg, Linz, Austria; 4 Ludwig-Boltzmann Institute for Experimental and Clinical Traumatology, Vienna, Austria; 5 Department of Orthopaedic Surgery, Ordensklinikum Linz Barmherzige Schwestern, Teaching Hospital of the Paracelsus Medical University Salzburg, Linz, Austria; 6 Department of Marketing and Electronic Business, University of Applied Sciences Upper Austria, Steyr, Austria; 7 Research Unit of Orthopedic Sports Medicine and Injury Prevention, Institute for Sports Medicine, Alpine Medicine and Health Tourism (ISAG), UMIT, Hall in Tirol, Austria; 8 Department of Radiology, Paracelsus Medical University, Salzburg, Austria; 9 Faculty of Health, Social Care and Education, Kingston University and St George’s, University of London, London, UK

**Keywords:** Return to sport, Athletic injuries, Anterior cruciate ligament injuries, Risk assessment

## Abstract

**Purpose:**

There is sparse evidence for return to sport criteria after knee injury. Functional performance deficits, particularly in fatigued muscular condition, should be verified prior to the attempt to return to high-risk pivoting sports. The purpose of this study was to generate reference values for the limb symmetry index (LSI) of healthy subjects in fatigued and non-fatigued muscular condition in a newly designed test battery.

**Methods:**

Forty-two healthy subjects [22 females, 20 males; mean (SD) age 30.4 (6.6) years] were evaluated using a test battery consisting of an isometric strength test, a series of five single-leg hop tests and an integrated fatigue protocol. Subjective physical activity was assessed with the Tegner Activity Scale (TAS). The cut-off values for healthy subjects were calculated considering the fifth percentile as the minimum reference value for the LSI and single-leg hop distance.

**Results:**

The mean (SD) overall LSI was 98.8% (4.6). No significant gender or age specific differences in limb symmetry were observed. The comparison of the non-fatigued LSI with the overall LSI revealed no clinically relevant change due to muscular fatigue. Repeated measures ANOVA revealed a significant within effect on fatigue/non-fatigue condition (*F*_(1,38)_ = 18.000; *p* < 0.001, *η* ^2^ = 0.321) on absolute single-leg hop distance. Moreover, a significant between effect on the TAS-parameter (*F*_(1,38)_ = 5.928; *p* = 0.020, *η* ^2^ = 0.135 between: TAS ≤ 5/TAS > 5) and on gender (*F*_(1,38)_ = 23.956; *p* < 0.001, *η* ^2^ = 0.387) could be detected.

**Conclusions:**

The absolute jumping distance in the single-leg hop for distance was significantly reduced due to fatigue. No clinically relevant effect of muscular fatigue was observed on limb symmetry in our study sample. Gender and physical activity are important factors to be considered when interpreting reference values.

**Electronic supplementary material:**

The online version of this article (10.1007/s11332-017-0410-5) contains supplementary material, which is available to authorized users.

## Introduction

Lesions of the anterior cruciate ligament (ACL) are the most common ligamentous injuries in the young and athletic population. The estimated number of ACL reconstructions is between 100,000 and 200,000 annually in the United States, with an approximate yearly incidence of 0.03–0.06% [1]. Seventy-seven percent of ACL insufficient knees result in permanent moderate to severe physical limitations [2].

Particularly problematic is the high ACL re-rupture rate. Up to 30% of young active patients who undergo ACL reconstruction suffer a second ACL rupture in the first 2 years after surgery in either the ipsilateral or contralateral knee [3–7]. A recent systematic review and meta-analysis demonstrated that younger age and a return to high level of activity are salient factors associated with secondary ACL injury [7]. Paterno et al. reported a six times greater overall incidence rate of a second ACL injury within 24 months after reconstruction compared to first-ever ruptures [3]. A factor that might contribute to such high ACL re-rupture rates is a premature return to sports activities [4]. In patients who fulfilled specific return-to-sport criteria, an estimated 84% decrease in knee re-injury rate has been reported [4]. It has been suggested that an objective determination of the right point in time for safe return to pivoting sports after ACL reconstruction is of utmost importance, in order to decrease the likelihood of re-injury related to premature return to sports [1, 8].

Several test protocols have been designed to provide such objective measures, which should facilitate deciding when a return to contact or high-risk pivoting sports may be considered safe [8, 9]. Test batteries consisting of single-leg hop tests have previously been described by different authors [1, 10–15], whereby a combination of at least two tests and testing under fatigued conditions is recommended to increase sensitivity in detecting abnormal limb symmetry [12].

However, little is known about the element of fatigue when performing single-leg hop tests, and how this may influence subsequent judgments about return to sport. Adding a fatigue protocol to the diagnostic battery represents a novel approach to the diagnostic investigation of the knee, as the risk of injury might increase with muscular fatigue. According to previous studies, kinematic changes due to fatigue result in higher impact accelerations, increasing the risk of overload injuries [16]. Injuries often tend to occur at the end of a sporting event, because fatigue-induced modifications in lower-limb control may increase the risk of noncontact ACL injury during landings [17]. To improve the sensitivity of tests for functional impairment after ACL reconstruction, the testing of dynamic function under fatigued conditions has been suggested previously [12]. The expectation is that testing in state of muscular fatigue will reveal a higher informative value for the evaluation of lower extremity functional performance, thereby enhancing the decision to release a person to pivoting sports and ultimately resulting in a lower risk of injury or re-injury.

Our study aimed to explore a novel diagnostic single-leg hop test battery with an integrated fatigue protocol in a group of healthy participants. For this, we composed a diagnostic battery from suitable previously described hop tests [1, 12–15, 18]. The most frequently reported objective criterion for assessing whether muscle strength and hop performance are normal or abnormal, is the Limb symmetry index (LSI). The underlying rationale is that this may minimize overuse or acute injury when returning to sports or strenuous work in patients who underwent knee surgery [11, 19].

Our objectives were (a) to assess LSI values of the new test battery in a healthy population; (b) to describe the LSI in fatigued and non-fatigued state for this group of participants; (c) to explore if the contextual factors age, gender and physical activity affect the LSI; (d) to compute reference values for the LSI and absolute jumping distances in a population considered as healthy.

## Methods

### Study design and participants

We conducted a prospective cross-sectional observational pilot study, for which we recruited a sample of 42 healthy subjects. The study was publicized at the host institution between December 2015 and October 2016.

Inclusion Criteria were (1) female and male subjects, (2) age between 16 and 60 years (epiphyseal fusion completed), (3) a self-assessed general level of fitness that allows completion of the physical components of the assessment.

Exclusion Criteria were (1) any kind of known previous injury of hip, knee and ankle joints in both limbs, (2) pregnant and nursing women, (3) concomitant medication or conditions that interfere with a person’s ability to comply with study procedures.

The study was registered on the ClinicalTrials.gov database (unique identifier: NCT02760589).

### Study procedures

All subjects were evaluated by the same examiner (IL) in a single testing session in a standardized test setting at the Trauma Center (Unfallkrankenhaus) Linz.

### Assessments

Subjects underwent the following assessments: (1) Tegner Activity Scale, (2) determination of limb dominance, and (3) a single-leg hop test battery with fatigue protocol.

#### Tegner Activity Scale (TAS)

The subjective physical activity level of all subjects was determined with the German Version of the Tegner Activity Scale (TAS) [20, 21]. The TAS is a self-administered questionnaire and describes the subjective physical activity of an individual on an 11-point ordinal scale (range 0–10), where higher score indicates higher level of activity. We included the TAS to describe sample characteristics, and also as a potential explanatory variable in correlation analysis.

#### Limb dominance

Limb dominance was established using three standard tests [22]. Dominance was set for the leg used for at least two out of the three following tests: First, subjects were asked with which leg they preferred to kick a ball. Second, subjects were asked to step onto a raised platform (25 cm height). Third, subjects were put off balance in standing, by the tester’s controlled push from behind between the shoulder blades. As an index of central tendency, mode was adopted for the determination of limb dominance.

#### Single-leg hop test battery with fatigue protocol

The novel single-leg hop test battery with integrated fatigue element was administered according to the following standardized protocol: Subjects were required to attend the test appointment in sports shoes. The test battery consists of a standardized warm-up protocol (10 min stationary cycling, 2 × 10 squats, 2 × 10 calf raises, ten jumps on both legs, five unilateral jumps each leg) followed by an isometric strength test (testing the maximum voluntary isometric contraction) of the hamstrings in prone position in 90 degree knee flexion using a portable dynamometer (Mecmesin Advanced Force Gauge, Mecmesin, UK), which is attached to a wall bar with a non-stretchable rope. Isometric hamstring force was assessed in this group of healthy subjects for the further comparison with patients who underwent ACL reconstruction to ascertain to which extent post-operative donor morbidity due to semitendinosus and gracilis tendon harvest affects hamstring force.

After that several single-leg jump tests are performed: (1) single-leg hop for distance (SLHD), (2) single-leg 6 m timed hop, (3) single-leg triple crossover hop for distance and (4) side hop test. The tests were chosen on the basis of hop tests commonly described in the literature [1, 10–15, 23]. Lastly, a fatigued single-leg hop for distance is conducted following a fatigue protocol. For the fatigue protocol subjects were asked to perform alternating squats lunges for the duration of 2 min continuously but at their own comfortable pace. If maximum voluntary exertion was reached within 2 min (i.e. unable to perform further squat lunges) participants were allowed to pause within the 2 min measurement but asked to restart as soon as possible. To the authors knowledge there is no comparable fatigue protocol described previously. It is hypothesized that muscle fatigue accumulates during the hop testing and the subsequent fatigue protocol, resulting in a higher significance in the achieved LSI values [16]. We have provided a detailed description of this test battery in an online supplemental file.

### Data analysis

#### Sample size determination

Because the present study is a pilot study, the authors refrained from a formal sample size and power calculation and opted for a convenience sample. We aimed to recruit an equal number of female and male participants.

#### Statistical methods

The LSI was calculated such that the score of the non-dominant leg is expressed as a percentage of the score of the dominant leg. LSI values were calculated for each single test (1) isometric strength test, (2) single-leg hop for distance, (3) 6-m timed hop, (4) triple crossover hop for distance, (5) side hop, and (6) fatigue single-leg hop for distance, and for the overall test battery as an average percentage.

The non-fatigued LSI was calculated as an average of the subtests from the isometric strength test and the four hop tests prior to the fatigue protocol. The fatigued LSI was calculated as an overall combination of all test items including the LSI for the fatigue single-leg hop for distance (fatigue-SLHD-LSI). The fatigued LSI represents the overall LSI of the test battery. We compared the non-fatigued LSI with the overall LSI in order to assess if limb symmetry changes due to muscular fatigue in healthy subjects. Although the overall LSI score was the primary outcome measure, the absolute scores on each battery item are also reported to better understand the behavior of the calculated overall LSI of the diagnostic battery. To evaluate the impact of the fatigue protocol on single leg hop performance, we compared the absolute values (i.e. the jumping distance in centimeters) of the single-leg hop for distance before and after the fatigue protocol. We used the Shapiro–Wilk-Test to confirm that LSI values were normally distributed.

Data were stratified into two TAS groups, two age groups, and according to gender. Spearman’s correlation coefficient was calculated to determine, whether subjective physical activity level (TAS*)* correlated with LSI values.

Non-fatigued LSI and overall LSI were analyzed by using a 2 (between: male/female) × 2 (between: TAS ≤ 5/TAS > 5) × 2 (within: non-fatigue/fatigue) repeated measures ANOVA to examine whether LSI differs depending on the gender, the TAS-Level or fatigue.

Mean SLHD and mean fatigue SLHD were also analyzed by using a 2 (between: male/female) × 2 (between: TAS ≤ 5/TAS > 5) × 2 (within: non-fatigue/fatigue) repeated measures ANOVA to examine whether the absolute jumping distance in the SLHD differs depending on the gender, the TAS-Level or fatigue.

Reference values were calculated for non-fatigued LSI, overall LSI, mean SLHD and mean fatigue SLHD. As a measure of central distribution both mean and standard deviation, median and fifth percentile were used, considering the fifth percentile as the minimum reference value. The fifth percentile value in each group should be considered as cut-off value for healthy subjects [24].

All statistical analyses were performed using SPSS statistical software (version 23, SPSS Inc., Chicago, Illinois, USA), where *p* < 0.05 indicated a statistically significant result and calculating 95% confidence intervals.

## Results

A sample of convenience of 42 healthy subjects (22 females and 20 males) participated in this study. The mean (SD) age was 30.4 (6.6) years (range 18–47). The median (IQR) TAS was 5.5 (3). No difference between female and male subjects was found in terms of the subjective level of physical activity (*p* < 0.05). The right leg was dominant in 37 (88.1%) cases and the left leg in 5 cases (11.9%). Participant characteristics are presented in Table 1.

**Table 1:** Participant characteristics (entire sample and by gender)

	Entire sample (*n* = 42)	Female (*n* = 22)	Male (*n* = 20)	p*
Age (years), mean (SD)	30.4 (6.6)	29.7 (7.1)	31.1 (6.1)	0.485
Height (m), mean (SD)	172.7 (8.9)	167 (6.5)	179.1 (6.6)	< 0.001
Weight (kg), mean (SD)	68.9 (15.7)	57.7 (6.3)	81.1 (13.7)	< 0.001
BMI (kg/m^2^)	22.9 (3.6)	20.7 (1.8)	25.2 (3.5)	< 0.001
TAS, median (IQR)	5.5 (3)	6 (3)	5 (3)	0.817
Leg dominance (left/right)	5/37	2/20	3/17	–
Non-fatigued LSI, mean (SD)	97.7 (4.4)	97.8 (4.6)	97.4 (4.2)	0.774
Overall LSI, mean (SD)	98.8 (4.6)	99.2 (4.9)	98.4 (4.4)	0.605
Mean SLHD (cm), mean (SD)	138.8 (31.4)	122.5 (25)	156.6 (28.3)	< 0.001
Mean fatigue SLHD (cm), mean (SD)	128.3 (30.1)	112.7 (21.2)	145.4 (29.4)	< 0.001

*TAS* Tegner Activity Scale, *non-fatigued LSI* mean LSI of the subtests prior to the fatigue protocol, *overall LSI* mean overall combination including the LSI in the fatigue single-leg hop for distance, *mean SLHD* mean value in the single-leg hop for distance at the beginning of the test battery, *mean fatigue SLHD* mean value in the fatigued single-leg hop for distance at the end of the test battery

* t test between gender

Normality of distribution of LSI and SLHD was determined using Shapiro–Wilk. See Table 2 for the results.

**Table 2:** Results of the Shapiro–Wilk-test

	Statistics	*df*	Significance
Non-fatigued LSI	0.961	42	0.163
Overall LSI	0.983	42	0.779
Mean SLHD (cm)	0.981	42	0.693
Mean fatigue SLHD (cm)	0.981	42	0.686

*non-fatigued LSI* mean LSI of the subtests prior to the fatigue protocol, *overall LSI* mean overall combination including the LSI in the fatigue single-leg hop for distance, *mean SLHD* mean value in the single-leg hop for distance at the beginning of the test battery, *mean fatigue SLHD* mean value in the fatigued single-leg hop for distance at the end of the test battery

The implementation of the hop test battery took approximately 50–70 min for each participant. All subjects were able to complete the entire test battery and no injuries occurred throughout the test procedure. The mean (SD) absolute values for the subtests of the test battery (isometric strength test and the five hop tests) are presented in Table 3 for males and females separately.

**Table 3:** Absolute values for every subtest in the test battery

	Female (*n* = 22)			Male (*n* = 20)		
	Dom	Non-dom	Mean^a^	Dom	Non-dom	Mean^a^
Iso (N), mean (SD)	124.9 (29.9)	120.7 (28.7)	122.8 (28.4)	189.1 (56.1)	182 (57.6)	185.6 (55.4)
SLHD (cm), mean (SD)	122.5 (25.8)	122.6 (24.5)	122.5 (25)	158.2 (29)	155.1 (28.1)	156.6 (28.3)
6 m TH (s), mean (SD)	2.4 (0.3)	2.5 (0.5)	2.4 (0.4)	1.9 (0.3)	1.9 (0.3)	1.9 (0.3)
3 COH (cm), mean (SD)	328 (85.6)	329.8 (79.8)	328.9 (81.4)	456.2 (106.6)	449.4 (112.6)	452.8 (107.5)
SH (reps), mean (SD)	36 (9.4)	34.1 (8.7)	35 (8.6)	50.8 (11.4)	49.2 (13.2)	50 (12.2)
Fatigue SLHD (cm), mean (SD)	111 (20.1)	114.4 (23.1)	112.7 (21.2)	145 (30.2)	145.9 (29.4)	145.4 (29.4)

*Dom* dominant leg, *Non-dom* non-dominant leg, *Iso* isometric hamstring strength, *SLHD* single-leg hop for distance, *6m TH* single-leg 6m timed hop, *3 COH* single-leg triple crossover hop for distance, *SH* repetitions in the 30 s side hop test, *fatigue SLHD* fatigued single-leg hop for distance

^a^ Mean combined results (of the dominant and non-dominant leg)

### Limb symmetry indices

The average overall LSI was 98.8% (4.6) mean (SD). There was no significant gender difference. The LSI values in percentage for every subtest are presented in Table 4. The LSI values for the overall test battery are presented in Table 1.

**Table 4:** Limb symmetry index (LSI) values in percentage for every subtest

	Entire sample (*n* = 42)	Female (*n* = 22)	Male (*n* = 20)	p*
LSI Iso, mean (SD)	96.7 (12.1)	97.2 (11.3)	96.3 (13.1)	0.809
LSI SLHD, mean (SD)	99.4 (5.1)	100.4 (5.3)	98.3 (4.7)	0.181
LSI 6 m TH, mean (SD)	95.8 (9.3)	93.9 (10.6)	98 (7.2)	0.159
LSI 3 COH, mean (SD)	100.1 (9.7)	101.5 (9.1)	98.5 (10.3)	0.321
LSI SH, mean (SD)	96.2 (13.7)	96.2 (17.2)	96.2 (8.7)	1.000
LSI fatigue SLHD, mean (SD)	102.1 (7.4)	102.9 (7.9)	101.1 (7)	0.442

*LSI Iso* LSI of the isometric hamstring strength test, *LSI SLHD* LSI of the single-leg hop for distance, *LSI 6m TH*  LSI of the single-leg 6m timed hop, *LSI 3 COH* LSI of the single-leg triple crossover hop for distance, *LSI SH* LSI of the 30 s side hop test, *LSI fatigue SLHD* LSI of the fatigued single-leg hop for distance

*t test between gender

### Effect of fatigue on limb symmetry

A 2 (between: male/female) × 2 (between: TAS ≤ 5/TAS > 5) × 2 (within: non-fatigue/fatigue) repeated measures ANOVA between the non-fatigued LSI and the overall LSI revealed a significant within effect on fatigue/non-fatigue condition (*F*_(1,38)_ = 4.457; *p* = 0.041, *η* ^2^ = 0.105, observed power = 0.16). Moreover, a significant between effect on the TAS-parameter (between: TAS ≤ 5/TAS > 5) could be detected (*F*_(1,38)_ = 4.582; *p* = 0.039, *η* ^2^ = 0.108). Gender as a further between factor did not reach significance (*F*_(1,38)_ = 0.075; *p* = 0.785, *η* ^2^ = 0.002). No interaction effects on both, within and between factors, could be detected.

### Effect of gender, age and TAS on limb symmetry

There was no significant difference between female and male subjects in terms of limb symmetry (Table 1). No significant difference was found between the two age groups (18–27 and > 27 years) for the non-fatigued LSI and the overall LSI (*p* < 0.05).

TAS correlated significantly with the non-fatigued LSI, with a Spearman’s rank coefficient of 0.326 (*p* = 0.035, 95% CI 0.02–0.65), representing a moderate to weak correlation. No significant correlation was found between TAS and the overall LSI.

### Effect of fatigue on absolute jumping distance

A 2 (between: male/female) × 2 (between: TAS ≤ 5/TAS > 5) × 2 (within: non-fatigue/fatigue) repeated measures ANOVA between the mean SLHD (jumping distance in cm) and the mean fatigue SLHD (cm) revealed a significant within effect on fatigue/non-fatigue condition (*F*_(1,38)_ = 18.000; *p* < 0.001, *η* ^2^ = 0.321). Moreover, a significant between effect on the TAS-parameter (*F*_(1,38)_ = 5.928; *p* = 0.020, *η* ^2^ = 0.135 between: TAS ≤ 5/TAS > 5) and on gender (*F*_(1,38)_ = 23.956; *p* < 0.001, *η* ^2^ = 0.387) could be detected. Also, no interaction effects could be revealed.

**Fig. 1:**
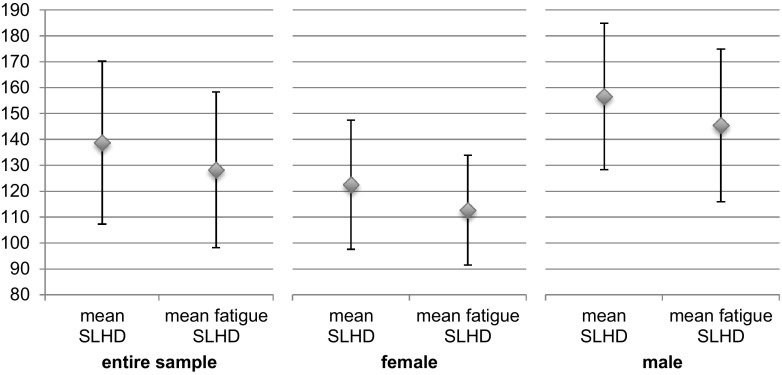
Mean absolute values in the single-leg hop for distance in non-fatigued and fatigued muscular condition. Error bars represent standard deviations

Paired *t* tests revealed a significant reduction in jumping distance for the single-leg hop for distance after the fatigue protocol (Fig. 1). The mean (SD) difference in jumping distance of the whole sample was 10.5 cm (15.6) (*p* < 0.001, 95% CI 5.6–15.4) between the single-leg hop for distance at the beginning of the test battery and at the end (i.e. after the fatigue protocol).

### Reference values

The cut-off values for healthy subjects were calculated considering the fifth percentile as the minimum reference value for (1) the non-fatigued LSI, (2), the overall LSI (3) the mean jumping distance in the single leg hop for distance and (4) the mean jumping distance in the fatigue single leg hop for distance. A subject is considered “healthy” if he or she reaches the minimum reference value in both, the LSI and the absolute jumping distances.

Suggested minimum reference values for overall LSI, non-fatigued LSI, mean SLHD and mean fatigue SLHD are presented as the 5th percentile in Tables 5, 6, 7 and 8, stratified by gender and TAS group.

**Table 5:** Non-fatigued LSI

	Male			Female		
	Mean (SD)	Median	P5*	Mean (SD)	Median	P5*
TAS (≤ 5)	96.91 (4.33)	97.12	87.60	95.01 (3.75)	95.46	85.31
TAS (> 5)	96.91 (4.28)	97.56	92.38	100.19 (3.87)	99.88	94.05

* Cut-off value for healthy subjects

**Table 6:** Overall LSI

	Male			Female		
	Mean (SD)	Median	P5*	Mean (SD)	Median	P5*
TAS ≤ 5	98.41 (4.27)	98.19	91.01	96.99 (3.23)	96.89	92.57
TAS > 5	98.45 (4.89)	96.60	91.79	101.01 (5.37)	102.05	89.94

* Cut-off value for healthy subjects

**Table 7:** Mean SLHD (cm)

	Male			Female		
	Mean (SD)	Median	P5*	Mean (SD)	Median	P5*
TAS ≤ 5	143.30 (26.57)	144.17	98.00	116.27 (19.52)	114.83	82.67
TAS > 5	172.91 (21.79)	177.17	145.50	127.72 (28.54)	132.75	76.83

*Mean SLHD* mean value in the single-leg hop for distance at the beginning of the test battery

* Cut-off value for healthy subjects

**Table 8:** Mean fatigue SLHD (cm)

	Male			Female		
	Mean (SD)	Median	P5*	Mean (SD)	Median	P5*
TAS ≤ 5	133.09 (27.88)	129.00	93.50	111.85 (10.51)	115.25	95.50
TAS > 5	160.50 (24.92)	151.00	129.50	113.33 (27.67)	113.50	64.00

*Mean fatigue SLHD* mean value in the fatigued single-leg hop for distance at the end of the test battery

* Cut-off value for healthy subjects

## Discussion

### Overall findings

According to previous studies the risk of lower leg injuries increases with muscular fatigue [12, 16]. We considered that testing lower limb performance under fatigued conditions might reveal higher informative value for the evaluation of functional performance. It is anticipated, that muscular fatigue accumulates during performing the test battery. The participants in this study indeed experienced a statistically significant decrease in jumping distance of approximately 10.5 cm in either leg in the single-leg hop for distance due to fatigue. These results indicate that the test battery that was composed for this study suited the requirements to induce muscular fatigue, resulting in a demonstrable deterioration in functional performance in healthy subjects. More importantly, the lower limb symmetry did not change with fatigue. These results suggest, that there is no effect of muscular fatigue on limb symmetry in healthy subjects. It may be hypothesized, that testing in state of muscular fatigue will reveal abnormalities in limb symmetry in not yet adequately rehabilitated subjects after ACL reconstruction. The test battery might therefore be a suitable method to prevent a premature return to pivoting sports ultimately resulting in a lower risk of re-injury.

#### Strengths and weaknesses of the study

The limitations of the current study include potential contributing factors that impact knee function during landing in single-leg hop tests, which were not addressed in the present study. A number of studies have indicated the importance of hip and trunk muscle strength and activation for lower extremity control and knee biomechanics [25, 26]. In this study, we did not analyze the impact of trunk muscle function or movement patterns of the hip and ankle joint, which also may influence knee overall function.

A strength in our methodology is the description of suggested reference values for LSI and SLHD using the 5th percentile. Others have emphasized how norms that are expressed as means have limited practical value in clinical decision-making [27], and different approaches have been described to account for this [28]. Reference values based on population percentiles could provide a convenient benchmark for clinical practice.

#### Bias

A limitation of this study is the potential for selection bias, as subjects participated voluntarily in the physically demanding test protocol and may therefore not reflect the normal population. This inherent selection bias should be considered when interpreting these results and using the reference values.

#### Strengths and weaknesses in relation to other studies

At this point, the classifications whether an individual has normal or abnormal limb symmetry is not entirely clear as prior studies have reported inconsistent cut-off values for the LSI. A difference of 15% or less has shown to represent normal variation, regardless of limb dominance or sporting levels [29]. In contrast, Herbst et al. recommended an LSI of 90% or more for the dominant leg and an LSI of 80% or more for the non-dominant leg to determine the time for a return to play after ACL reconstruction [9]. Furthermore, it is not clear whether normal or abnormal LSI values are associated with a subject’s overall functional ability. Numerous previous studies have recommended that an LSI ≥ 90% may be classified as normal. [11, 12, 30] This study represents the first report of limb symmetry in healthy subjects using a battery of hop tests in order to generate reference values for clinical practice. The reference values obtained in our study deviate in part from previous recommendations on LSI cut-off values. This indicates that the currently recommended cut-off value of 90% is only restrictedly suitable. Further research with larger sample sizes should focus on the impact of physical activity on limb symmetry.

The use of the TAS for the determination of the level of physical activity may not be a suitable measurement method. Participants in this study demonstrated major differences in absolute jumping distances, which were not associated with the TAS. Since it is a subjective measure, some subjects may have either over- or underestimated their true level of physical activity. This has also been observed in a previous study, in which TAS scores showed no relationship with muscle strength 5 years after ACL reconstruction [31].

#### Practical implications and further research

Hop test scores should be evaluated based on normative data that are specific to the individual’s sex, age and level of physical activity. Our study has made a contribution to generating these data. To the authors’ knowledge, normative data for hop test performance in adults according to varying levels of physical activity are currently not available.

The clinical utility of the test battery to objectively determine the right point in time for a safe return to high-risk pivoting sports after ACL reconstruction should be investigated further. The aim of the present study was to present reference values for a population considered as healthy, so that these values can later be used to identify variations in patients after ACL injury. We will address this aspect in a parallel study, in which we will evaluate patients after ACL rupture 12–18 months after reconstruction and compare data with the results of the healthy control subjects in this study.

## Conclusions

The test battery that was composed for this study suited the requirements to induce muscular fatigue in healthy subjects. The participants experienced a statistically significant decrease in jumping distance in either leg in the single-leg hop for distance due to fatigue. The comparison of the non-fatigued LSI with the overall LSI revealed no clinically relevant change in lower limb symmetry due to muscular fatigue in our study sample of healthy subjects. Gender and physical activity are important factors to be considered when interpreting reference values. A subject is considered “healthy” if he or she reaches the minimum reference value in both, the LSI and the absolute jumping distance.

## Electronic supplementary material

Below is the link to the electronic supplementary material.

Supplementary material 1 (DOCX 1705 kb)

## Abbreviations

ACL: Anterior cruciate ligament
LSI: Limb symmetry index
RTS: Return To Sport
fatigue-SLHD-LSI: Limb symmetry index in the single-leg hop for distance in state of muscular fatigue
TAS: Tegner Activity Scale
UKH: Trauma Center
AUVA: Austrian Social Insurance for Occupational Risks
SLHD: Single-leg hop for distance
