# Supplementary material for: Reference values for fatigued versus non-fatigued limb symmetry index measured by a newly designed single-leg hop test battery in healthy subjects: a pilot study
Source: Sport Sci Health. 2017 Nov 10;14(1):105–13. doi: 10.1007/s11332-017-0410-5 (PMC5866266; doi:10.1007/s11332-017-0410-5)
Supplement: Supplementary file 1 — Supplementary material 1 (DOCX 1705 kb) [file 11332_2017_410_MOESM1_ESM.docx]

# Supplementary Material

1. Single-leg hop for distance: The subjects stand on the test leg and then hop as far as possible and land on the same leg. Free leg and arm swing is allowed (Figure 1). The subjects are instructed to perform a controlled, balanced landing and to keep the landing foot in place (i.e. no extra hops are allowed) until the examiner can register the landing position. The distance is measured in centimeters from the first toe at the push-off to the heel at the landing position.[1–12]

**Figure 1** Single-leg hop for distance


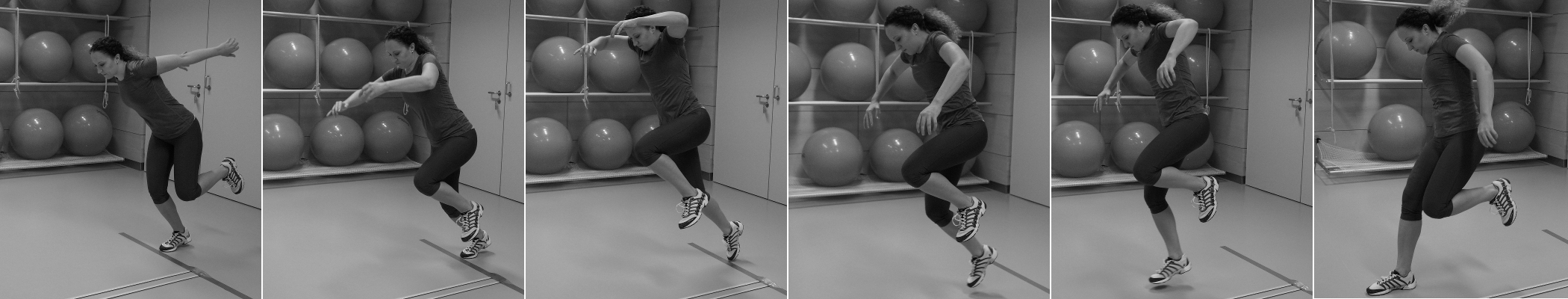


1. Single-leg 6 meter timed hop: Subjects are instructed to perform one-legged hops in series over the total distance of six meters (Figure 2). A standard stopwatch is used to record time. The stopwatch is started simultaneously with a verbal starting signal from the examiner and is stopped when the subject crosses the finishing line at six meters. The time is measured in seconds.[4, 8, 9, 11]

**Figure 2** Single-leg 6 meter timed hop


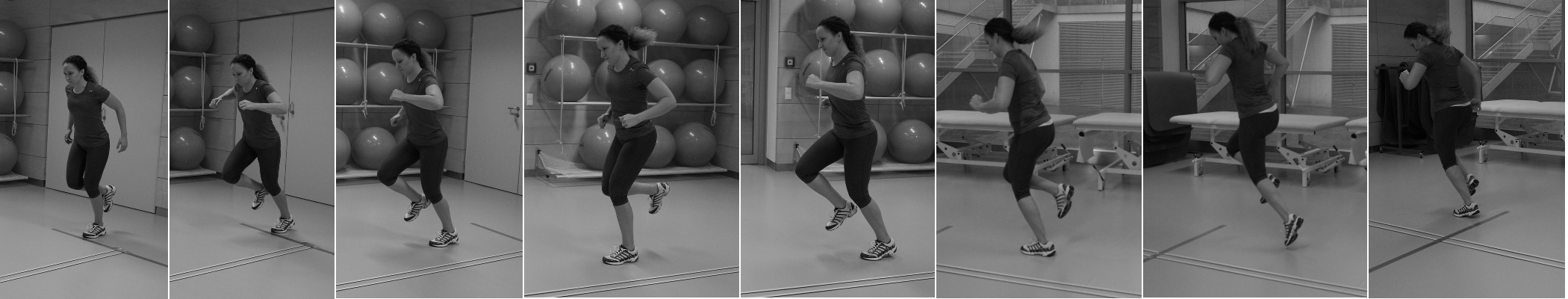


1. Single-leg triple crossover hop for distance: Subjects are instructed to hop forward three times while alternately crossing over two marking stripes, which are ten centimeters apart (Figure 3). The total distance hopped forward is recorded from the first toe at the push-off to the heel at the landing position. Subjects are instructed to position themselves at the left side of the stripes at the beginning of the hop on the left leg and vice versa for the right leg. The subjects are instructed to perform a controlled, balanced landing and to keep the landing foot in place (i.e. no extra hops are allowed) until the examiner can register the landing position. The distance is measured in centimeters.[4, 9–12]

**Figure 3** Single-leg triple crossover hop for distance


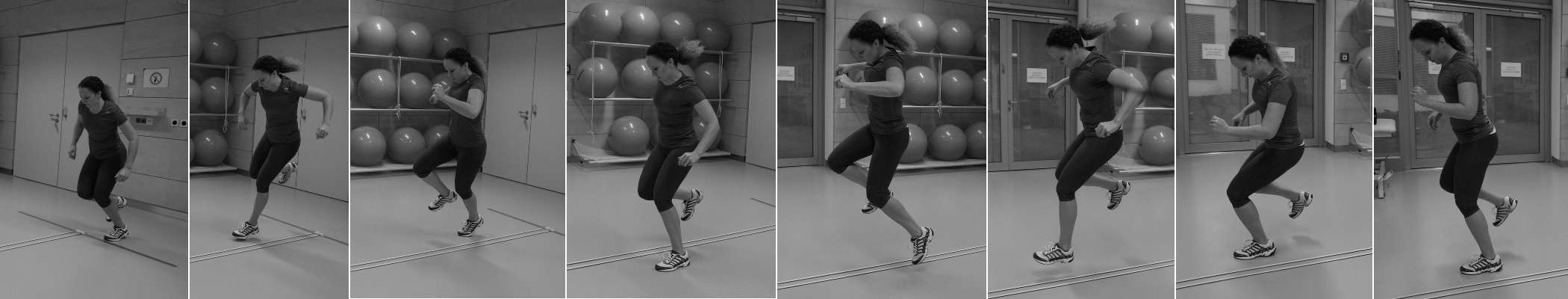


1. Side hop test: For the side hop test, the subjects stand on the test leg and jump from side to side between two parallel strips of tape, placed 40 cm apart on the floor (Figure 4). The subjects are instructed to jump as many times as possible during a period of 30 seconds. The number of successful jumps performed, without touching the tape with either foot or touching the floor with the contralateral foot, is recorded. A standard stopwatch is used to record time.[1, 3, 5, 7]

**Figure 4** Side hop test


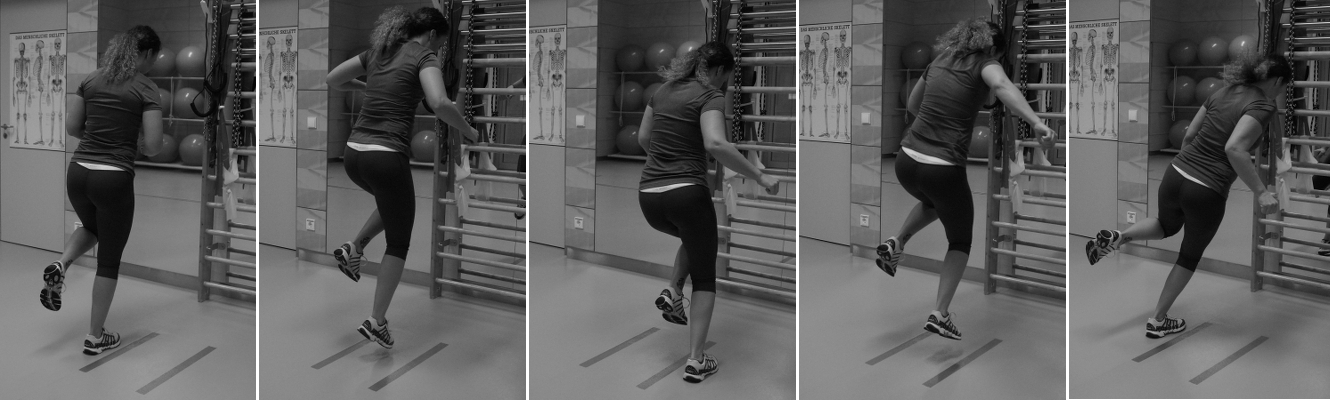


1. Fatigue single-leg hop for distance (Figure 1) is conducted following the fatigue protocol, which consists of alternating squat lunges to exhaustion for the duration of two minutes.

The subjects perform at least three practice trials followed by three maximum approved trials for the single-leg hop for distance and at least one practice trial followed by two maximum approved trials for the single-leg 6m timed hop and the single-leg triple crossover hop for distance. The side hop test is tested once. The mean of the valid trials for each leg in each test is used for data analysis.

An unsuccessful hop is classified for all hop tests by any of the following: touching down of the contralateral lower extremity, touching down of either upper extremity, loss of balance or an additional hop on landing. The number of invalid hops is documented.

Measurements from each item of the test battery were recorded on a case report form. All scores were recorded as absolute distance (centimeters), time (seconds), or repetitions in a given time period. The number of invalid trials for each subtest was also recorded.

# References

1. Gustavsson A, Neeter C, Thomeé P, et al (2006) A test battery for evaluating hop performance in patients with an ACL injury and patients who have undergone ACL reconstruction. Knee Surgery, Sport Traumatol Arthrosc 14:778–788. doi: 10.1007/s00167-006-0045-6

2. Manske R, Reiman M (2013) Functional performance testing for power and return to sports. Sports Health 5:244–50. doi: 10.1177/1941738113479925

3. Itoh H, Kurosaka M, Yoshiya S, et al (1998) Evaluation of functional deficits determined by four different hop tests in patients with anterior cruciate ligament deficiency. Knee Surg Sports Traumatol Arthrosc 6:241–245. doi: 10.1007/s001670050106

4. Barber-Westin SD, Noyes FR (2011) Factors used to determine return to unrestricted sports activities after anterior cruciate ligament reconstruction. Arthrosc - J Arthrosc Relat Surg 27:1697–1705. doi: 10.1016/j.arthro.2011.09.009

5. Thomeé R, Kaplan Y, Kvist J, et al (2011) Muscle strength and hop performance criteria prior to return to sports after ACL reconstruction. Knee Surgery, Sport Traumatol Arthrosc 19:1798–1805. doi: 10.1007/s00167-011-1669-8

6. Augustsson J, Thomeé R, Karlsson J (2004) Ability of a new hop test to determine functional deficits after anterior cruciate ligament reconstruction. Knee Surgery, Sport Traumatol Arthrosc 12:350–356. doi: 10.1007/s00167-004-0518-4

7. Thomeé R, Neeter C, Gustavsson A, et al (2012) Variability in leg muscle power and hop performance after anterior cruciate ligament reconstruction. Knee Surgery, Sport Traumatol Arthrosc 20:1143–1151. doi: 10.1007/s00167-012-1912-y

8. Harris JD, Abrams GD, Bach BR, et al (2014) Return to sport after ACL reconstruction. Orthopedics 37:e103-8. doi: 10.3928/01477447-20140124-10

9. Abrams GD, Harris JD, Gupta AK, et al (2014) Functional Performance Testing After Anterior Cruciate Ligament Reconstruction: A Systematic Review. Orthop J Sport Med 2:1–10. doi: 10.1177/2325967113518305

10. Björklund K, Andersson L, Dalén N (2009) Validity and responsiveness of the test of athletes with knee injuries: the new criterion based functional performance test instrument. Knee Surgery, Sport Traumatol Arthrosc 17:435–445. doi: 10.1007/s00167-008-0674-z

11. Reid A, Birmingham TB, Stratford PW, et al (2007) Hop testing provides a reliable and valid outcome measure during rehabilitation after anterior cruciate ligament reconstruction. Phys Ther 87:337–350.

12. Ardern CL, Webster KE, Taylor NF, Feller JA (2011) Return to the Preinjury Level of Competitive Sport After Anterior Cruciate Ligament Reconstruction Surgery: Two-thirds of Patients Have Not Returned by 12 Months After Surgery. Am J Sports Med 39:538–543. doi: 10.1177/0363546510384798
